# Stable N-doped & FeNi-decorated graphene non-precious electrocatalyst for Oxygen Reduction Reaction in Acid Medium

**DOI:** 10.1038/s41598-018-22114-1

**Published:** 2018-02-28

**Authors:** Nasser A. M. Barakat, Ahmed G. El-Deen, Zafar Khan Ghouri, Saeed Al-Meer

**Affiliations:** 1 Organic Materials and Fiber Engineering Department, Chonbuk National University, Jeonju, 561-756 Republic of Korea; 2 Chemical Engineering Department, Faculty of Engineering, Minia University, 61111 Minia, Egypt; 3 Materials science and Nanotechnology Department, Faculty of postgraduate Studies for Advanced Sciences, Beni Suef, Egypt; 4 Central Laboratory Unit, Qatar University, P. O. Box:, 2713 Doha, Qatar

## Abstract

NiFe nanoparticles-decorated & N-doped graphene is introduced as an effective and stable non-precious electrocatalyst for ORR in the acid medium. Compared to conventional Pt/C electrodes under the same conditions, the proposed nanocatalyst shows closer onset potential and current density. Typically, the observed onset potentials and current densities for the synthesized and Pt/C electrodes are 825 and 910 mV (vs. NHE) and −3.65 and −4.31 mA.cm^−2^ (at 5 mV.s^−1^), respectively. However, the most important advantage of the introduced metallic alloy-decorated graphene is its distinct stability in acid medium; the retention in the electrocatalytic performance after 1,000 successive cycles is approximately 98%. This finding is attributed to the high corrosion resistance of the NiFe alloy. The kinetic study indicates that the number of the transferred electrons is 3.46 and 3.89 for the introduced and Pt/C (20 wt%) electrodes, respectively which concludes a high activity for the proposed nanocomposite. The suggested decorated graphene can be synthesized using a multi-thermal method. Typically, nickel acetate, iron acetate, graphene oxide and urea are subjected to MW heating. Then, sintering with melamine in an Argon atmosphere at 750 °C is required to produce the final electrocatalyst. Overall, the introduced NiFe@ N-doped Gr nanocomposite shows remarkable electrochemical activity in the acid medium with long-term stability.

## Introduction

Undoubtedly, the confirmed near depletion of fossil oils is forcing the researchers to find effective, low cost, and environmentally safe energy devices. In addition to this dilemma, the serious negative impact to the climate promotes the elimination of the fossil fuels utilization. Flexibility, ease of management, and applicability make fuel cells the most promising energy devices to overcome the aforementioned problems. For example, wide utilization of the proton exchange fuel cells (PEMFCs) was one of the closing remarks of the 2015 Paris Climate Conference (COP21).

The main constituents of any PEMFC are anode, cathode, and membrane. Typically, oxygen is reduced at the surface of an active cathode while the fuel (e.g., hydrogen or alcohol) is oxidized at the anode surface^1^. Although there are numerous efforts to develop new membranes for the alkaline media fuel cells^2^, commercially, the acidic solutions-based PEMFCs are prevailing due to the excellent performance of the Nafion membrane. However, working in an acid medium limits the selection of the electrode materials as most of the metals show low corrosion resistance to acids. Accordingly, precious metals (e.g., Pt, Ru, and Pd) became inevitable choices for manufacturing the commercial electrode materials^3^. In acid media, platinum (Pt) is the most effective catalyst for both hydrogen oxidation and oxygen reduction^4,5^. In addition to Pt, palladium-based compounds also have considerable activity^6,7^. Though, the high cost and rarity of the precious metal electrodes are constraining the wide application of the PEMFCs^8^.

Disregarding the stability, many non-precious materials have shown good performance as anodes^9^. Recent research has indicated that bimetallic nanoparticles containing a first-row transition metal possess magnetic, optical, composition-dependent and catalytic properties that are different from mono metallic nanoparticle components^10^.

Considering that the most effective non-precious ORR catalysts are primarily nitrogen-doped nanocarbons (e.g. N-doped carbon nanotubes (CNT)^11^ and CNT/graphene mixture^12^), supporting of effective bimetallic nanoparticles on a proper N-doped carbonaceous material can distinctly enhance the ORR electrocatalytic activity^13^. Besides enhancing the electrocatalytic activity, N-doped carbon nanostructural supports showed more stability than nitrogen-free ones due to the high number of surface nucleation sites, which allows for anchorage and high dispersion of the catalyst nanoparticles on surface of the support material^14–16^. Moreover, due to the strong electron donor behavior of nitrogen, the doping process improves the durability of the produced carbon-support catalysts because of enhancement of π bonding^17,18^ and their basic properties^19^.

Graphene is a charming carbon material with excellent characteristics including a large theoretical surface area (2675 m^2^ g^−1^), strong mechanical strength, and excellent electrical conductivity. Consequently, it was exploited to enhance the performance of several promising electrode materials in the electrochemical devices^20–22^. Moreover, compared to other carbon nanostructures, the chemical route for graphene synthesis provides a good chance for functionalization by active groups which aids in decoration surface by metallic nanoparticles^9^.

The main target of this study is synthesis effective and highly stable non-precious bimetallic nanoparticles supported on nitrogen-doped graphene sheets to be exploited as electrocatalyst for the ORR in the acid media. It is known that the alloy structure of the transition metals can not only improve the catalytic activity of the final product but also may distinctly enhance the stability in the basic and acid media. Among the widely utilized transition metals in the electrochemical applications, Fe and Ni have very good contribution^23,24^.

In this study, we introduce NiFe alloy nanoparticle-decorated and N-doped graphene as a novel, stable, and efficient electrocatalyst for ORR in an acid medium. The proposed electrocatalyst was designed based on the following criteria: (1) Exploiting the alloy structure of the transition metals to enhance the stability in the acid media. (2) Maximizing the nitrogen content in the carbonaceous support to strength the catalyst activity toward the ORR. (3) Synthesis of the graphene sheets supports by the chemical route to develop chemical anchors for the transition metals nanoparticles which strongly improves the attachment and consequently ameliorates the activity of the final catalyst. Overall, the obtained results indicated that the proposed nanocomposite have very good electroactivity toward ORR with distinct stability in the acid media.

## Results and Discussion

### Catalyst characterization

**Figure 1:**
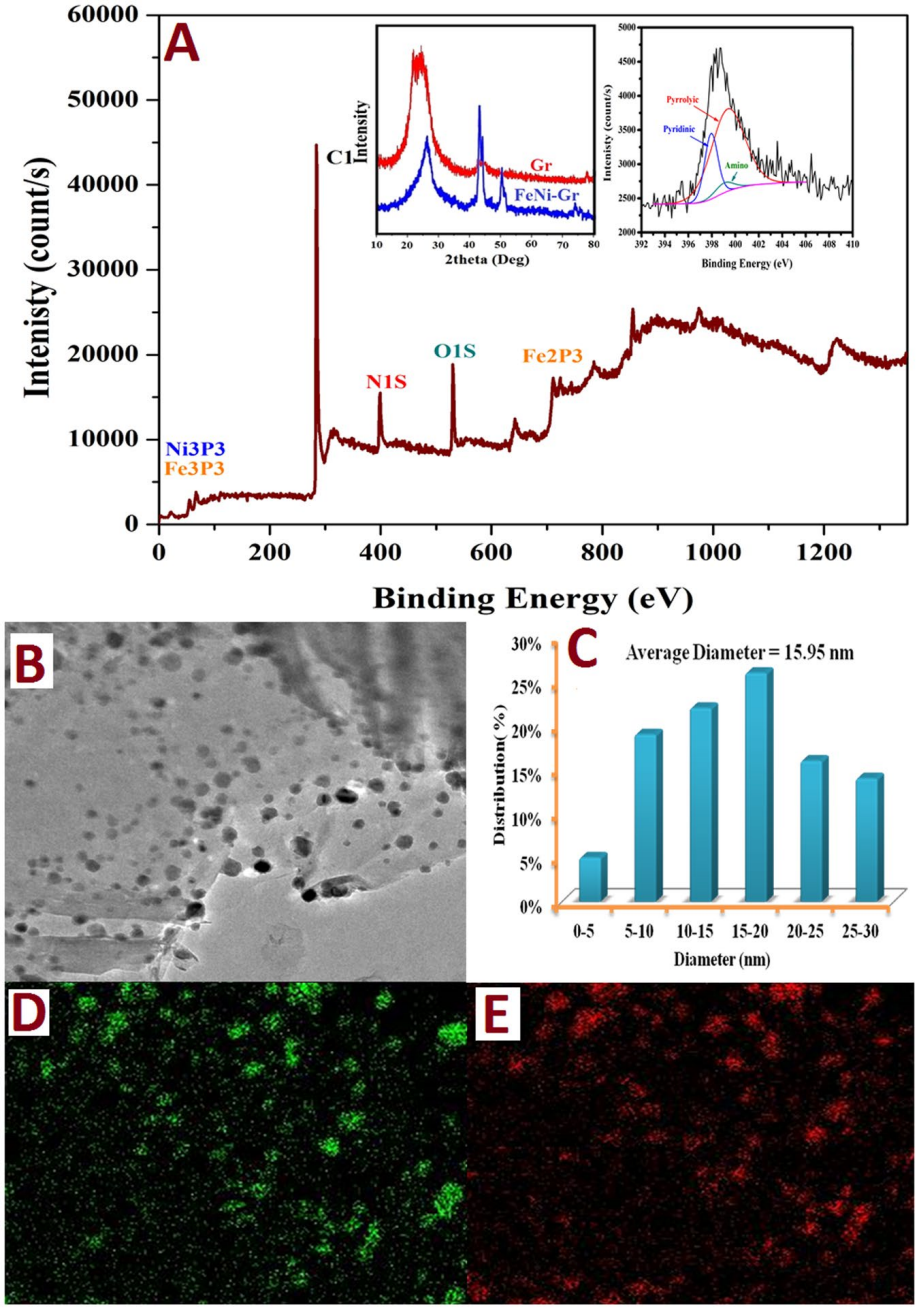
(**A**) XPS spectra for the FeNi@ N-doped graphene after the sintering process. Inset (left) demonstrates the analysis of N1S peak in the XPS spectrum. Inset (right) displays the XRD patterns for the pristine and bimetallic nanoparticles-decorated graphene, (**B**) TEM image, (**C**) size distribution of the metallic nanoparticles, and elemental mapping images (**D**; nickel and **E**; iron).

The XRD analysis can be used to confirm the graphene preparation. In other words, XRD analysis can differentiate between the graphite, graphene oxide and graphene. First, the graphite is usually assigned by a sharp peak at the 2θ value of 26.5°, which is indexed to the [002] crystal plane^25^. However, after the violent oxidation, the graphite peak disappears and a new diffraction peak appears at 2θ of 10.5° ^25^. On the other hand, due to the reduction process, the reduced graphene oxide shows a broad peak that can be fitted using a Lorentzian function into three peaks, which are centered at 2θ = 20.17°, 23,78° and 25.88°, which correspond to the interlayer distances of 4.47, 3.82 and 3.53 Å, respectively. These XRD results are related to the exfoliation and reduction processes of GO and the processes of removing intercalated water molecules and the oxide groups^26,27^. The observed broaden peak indicates the smaller crystalline size of graphene in the single-layer or few-layer structure. Accordingly, from left inset (Fig. 1(A)) which displays the XRD pattern of a pristine and decorated graphene sheets, one can claim that the pattern corresponding to the pristine graphene confirm the successful preparation of multiwall graphene sheets.

Accordingly, the prepared GO was used to prepare the proposed FeNi-decorated & N-doped graphene as it was explained in the experimental section. The XRD pattern of the synthesized composite is displayed in the left inset (Fig. 1(A)). The observed broad diffraction peak at 22.2~26.8° explains the graphene sheets disordered stacking. On the other hand, the three characteristic diffraction peaks corresponding to (111), (200) and (220) crystal planes at 2theta values of 43.8°, 51.1°, and 75.6°, respectively, confirm formation of the FeNi alloy (#47-1417)^28^. Based on XRD data base (#06-0696; Fe and #04-0850; Ni), iron and nickel are identified by the standard peaks at 2θ of 44.67°, 65.02° and 82.33°, and 44.05°, 54.85° and 76.37° corresponding to (110), (200) and (211), and (111), (200) and (220), respectively which indicates that some nanoparticles have a physical Fe/Ni mixture. Additionally, no peaks attributed to oxides or carbides were detected. According to the utilized characterizations which confirmed formation of pure Fe and Ni and considering the difficulty of evaporation of these metals due to the high melting points (Fe; 1538 °C, and Ni; 1455 °C), the weight of metals in the final produce can be estimated. Moreover, it was reported that calcination of the graphene oxide prepared by a similar chemical route to 750 °C leads to lose around 60 wt%^29^. Consequently, the Ni:Fe:C ratio in the produced composite can be determined to be 41:40:19 wt%, respectively.

Although XRD is highly trustable analytical technique, its utilization is limited to the crystalline materials. Therefore, to investigate nitrogen doping, X-ray photoelectron spectroscopy (XPS) was exploited. The obtained XPS spectra (Fig. 1(A)) indicate the successful nitrogen doping with a corresponding contents of 10.1%; this percentage has been further confirmed by FE-SEM EDX and elemental analysis (data are not shown). Moreover, the right inset (Fig. 1(A)) which displays the high-resolution of the N1s spectra indicates presence of N atoms with three different binding energies. These results depict that there are at least three typical nitrogen states in the introduced decorated graphene: amino (ca.399.05 eV), pyridinic (ca.398 eV) and pyrrolic (ca.399.63 eV)^30^.

Figure 1(B) describes the TEM image of the introduced FeNi@ N-doped graphene. As shown in Fig. 1(C), the average diameter of the metallic NPs distributed on the graphene sheets was 15.9 nm. Notably, elemental mapping was achieved to detect Fe and Ni distribution in a randomly selected graphene sheet. As shown in Fig. 1(D and E), nickel and iron have similar distribution, which affirms the mentioned hypothesis about the formation of metallic alloy (FeNi) nanoparticles.

### Electrochemical measurements

**Figure 2:**
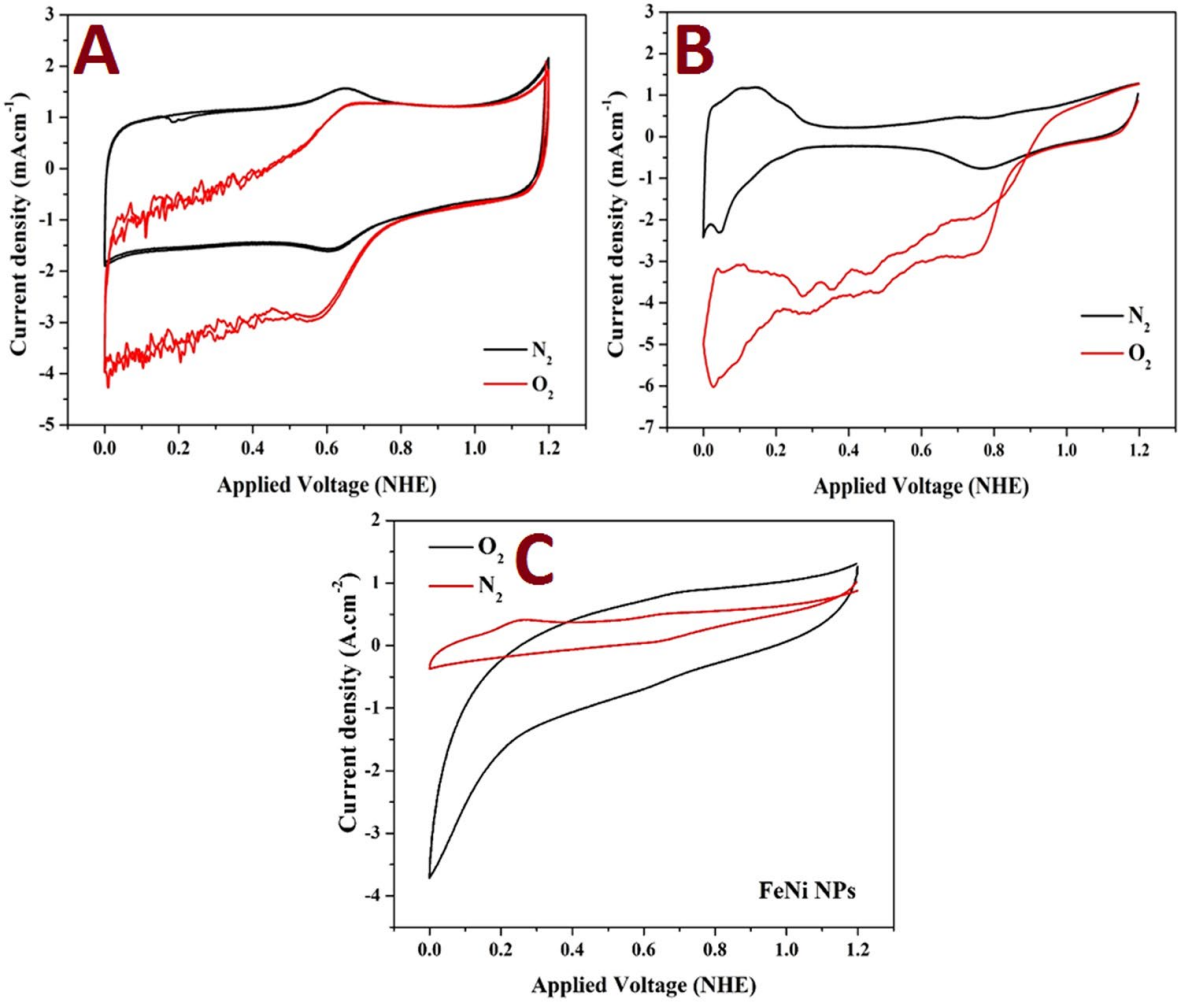
ORR activity at 5 mV.s^−1^ in 0.5 M H_2_SO_4_ for the (**A**) introduced FeNi-decorated and N-doped graphene, (**B**) Pt/C (20 wt%), and (**C**) FeNi NPs.

The electro-catalytic activity of the synthesized FeNi@ N-doped graphene sheets toward ORR was investigated by the corresponding current density and onset potential. Figure 2 displays the cyclic voltammograms of the introduced FeNi-N-Gr and conventional Pt/C (20 wt% Pt) in nitrogen- and oxygen- saturated 0.5 sulfuric acid solutions at 5 mV.s^−1^ scan rate and room temperature. The synthesized electrode reveals comparable performance with the precious metal electrode in the form of current density and onset potential. Based on previous reports, the onset potential is defined as the potential at which the background subtracted current density is equal to 0.1 mA.cm^−2^ ^31,32^. The onset potential for the introduced decorated graphene was close to that of Pt/C (Fig. 2B). Typically, the detected onset potentials were 725 and 810 mV (vs. RHE) for the modified graphene and Pt/C, respectively. Alternatively, the observed current densities were −3.65 and −4.31 mA/cm^2^ for the introduced and precious metal electrodes, respectively. Moreover, as shown in the figure, the oxygen adsorption process was a control step for the precious electrode, while the relatively good stability of the current density in the case of the introduced electrode indicates good oxygen adsorption affinity for the introduced catalyst. Specifically, the first step in the ORR process was adsorption of the molecular oxygen on the surface of the electrocatalyst^33,34^. As it was reported in literature, the oxygen reduction process in the acid media can occur by several pathways; direct reduction of hydrogen ions or formation of hydrogen peroxide intermediate^33,34^.

Regardless the reaction pathway, oxygen adsorption is the first step. Based on the results obtained in Fig. 2A, the indirect pathway is more likely to happen. It is worth mentioning that the oxygen adsorption step does not always distinctly affect the reaction rate. Accordingly, to investigate the influence of graphene support as well as to check the influence of oxygen adsorption step, FeNi nanoparticles have been prepared in absence of GO, urea and melamine. The electrocatalytic activity of the synthesized nanoparticles toward ORR is displayed in Fig. 2C. As shown, the unsupported nanoparticles have good activity toward hydrogen evolution reaction (HER), however they possess relatively low activity toward ORR compared to the supported ones. This finding emphasizes the role of the graphene support which can be attributed to the adsorption of both of hydrogens ions and oxygen molecules based on the known good adsorption capacity of the carbonaceous materials. At around 0.6 V, there is a pair of redox peaks can be observed in case of the introduced composite (Fig. 2A) which cannot be seen in case of the unsupported bimetallic nanoparticles (Fig. 2C). Considering the XRD analysis explains that there are unalloyed Fe and Ni in case of the proposed composite, the observed redox peaks can be assigned to these free metals. On the other hand, in case of the unsupported nanoparticles, well alloying process was conducted so no peaks could be observed. This pair of the redox peaks was almost at the same potential which indicates very good reversibility.

**Figure 3:**
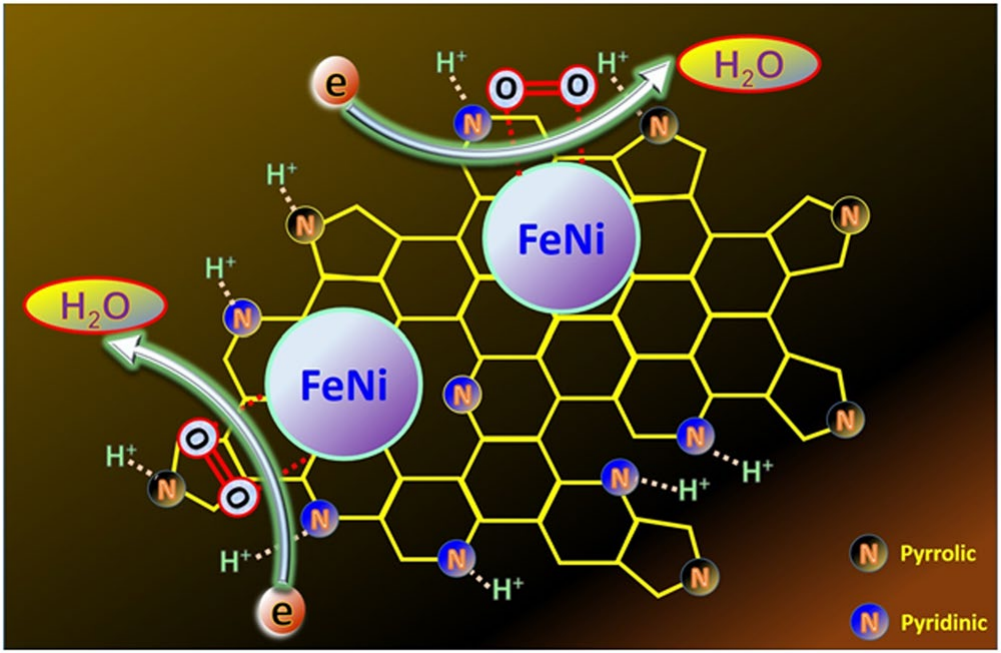
Schematic diagram for the oxygen reduction mechanism using the synthesized FeNi- N-Gr.

Doping the carbon nanostructures with heteroatoms such as N can distinctly change the properties of carbon. For instance, doping of carbon by nitrogen strongly enhances the oxidation resistance capability and its ORR catalytic activity. For example, doping of carbon nanofibers by nitrogen led to increase the onset potential of the oxygen reduction reaction by 70 mV with a corresponding electron transfer number of approximately 4^18^. It was concluded that, in the N-doped carbon nanostructures, the active sites are placed on the carbon atoms adjacent to the nitrogen atom^35^. Similarly, in the introduced electrocatalyst, the detected pyridinic and pyrrolic nitrogen (inset(right)), Fig. 1A) have a distinct role in the adsorption of hydrogen ions because of the strong tendency of nitrogen for electron donating^19^, while the metallic nanoparticles have more adsorption capacity for the molecular oxygen. Delivery of electrons, from the anode to the cathode, leads to complete the oxygen-hydrogen combination to form water molecules; ORR reaction. From the chemistry point of view, the pyridinic nitrogen has more basic characteristic than pyrrolic because the nitrogen lone pair electrons do not share in the pyridine cycle resonance. However, in case in pyrrole, the lone pair electrons contribute in the cycle resonance which negatively affects the basic characteristic. Accordingly, it is expected that pyridinic nitrogen has more attraction capacity for the hydrogen ions that reflects more contribution in the ORR reaction. It is noteworthy mentioning that the expected adsorption influence of the utilized N-doped graphene support can partially enhance the electrocatalytic activity of the proposed composite. However, the main impact can be assigned to avoiding the agglomeration of unsupported bimetallic nanoparticles which strongly improves a very important parameter in the heterogeneous catalytic reactions; contact area between the reactants and the catalyst surface. Furthermore, the excellent electrical conductivity of the utilized support has also a regarded effect. The proposed mechanism of O_2_ reduction on the surface of the introduced catalyst is visualized in Fig. 3.

**Figure 4:**
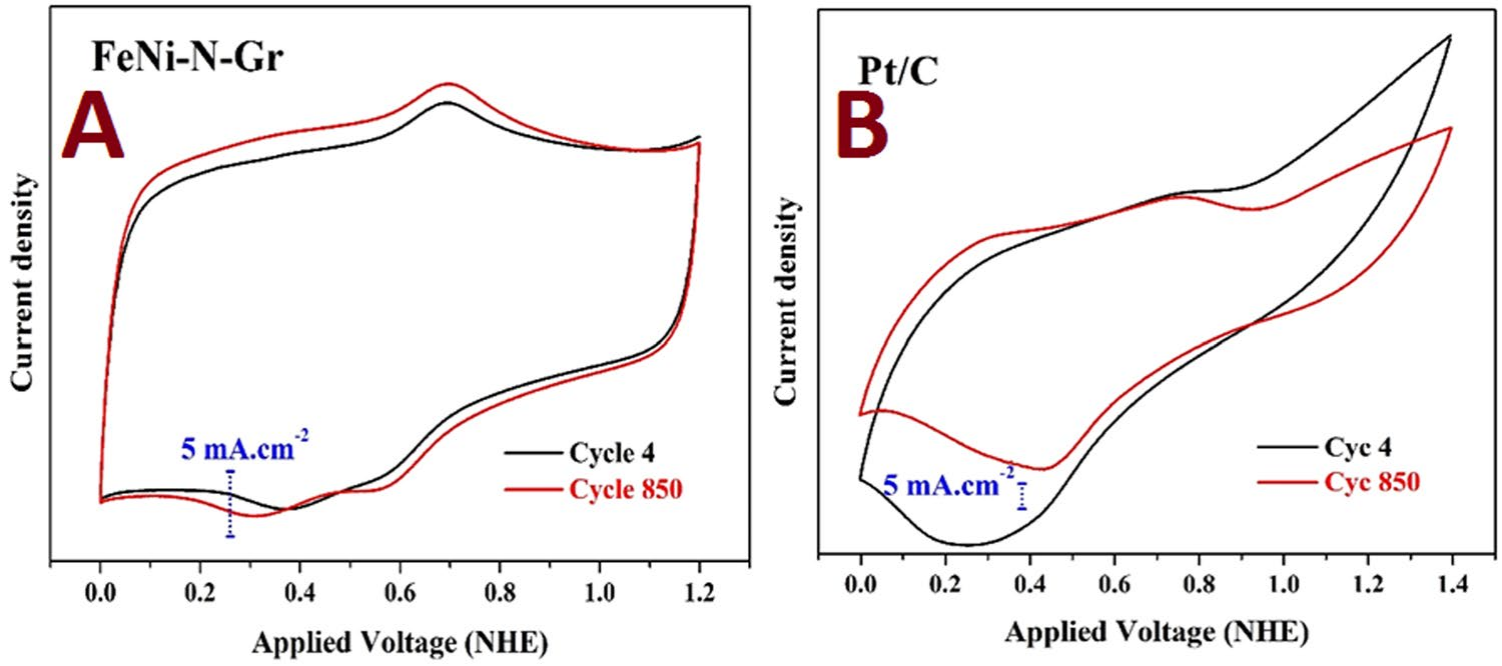
Comparison between the 4^th^ and 850^th^ cycles for the (**A**) introduced FeNi- N-Gr and (**B**) Pt/C electrodes. The data were extracted from 1,000 cycles in 0.5 M H_2_SO_4_ (scan rate 50 mV.s^−1^ at room temperature).

**Figure 5:**
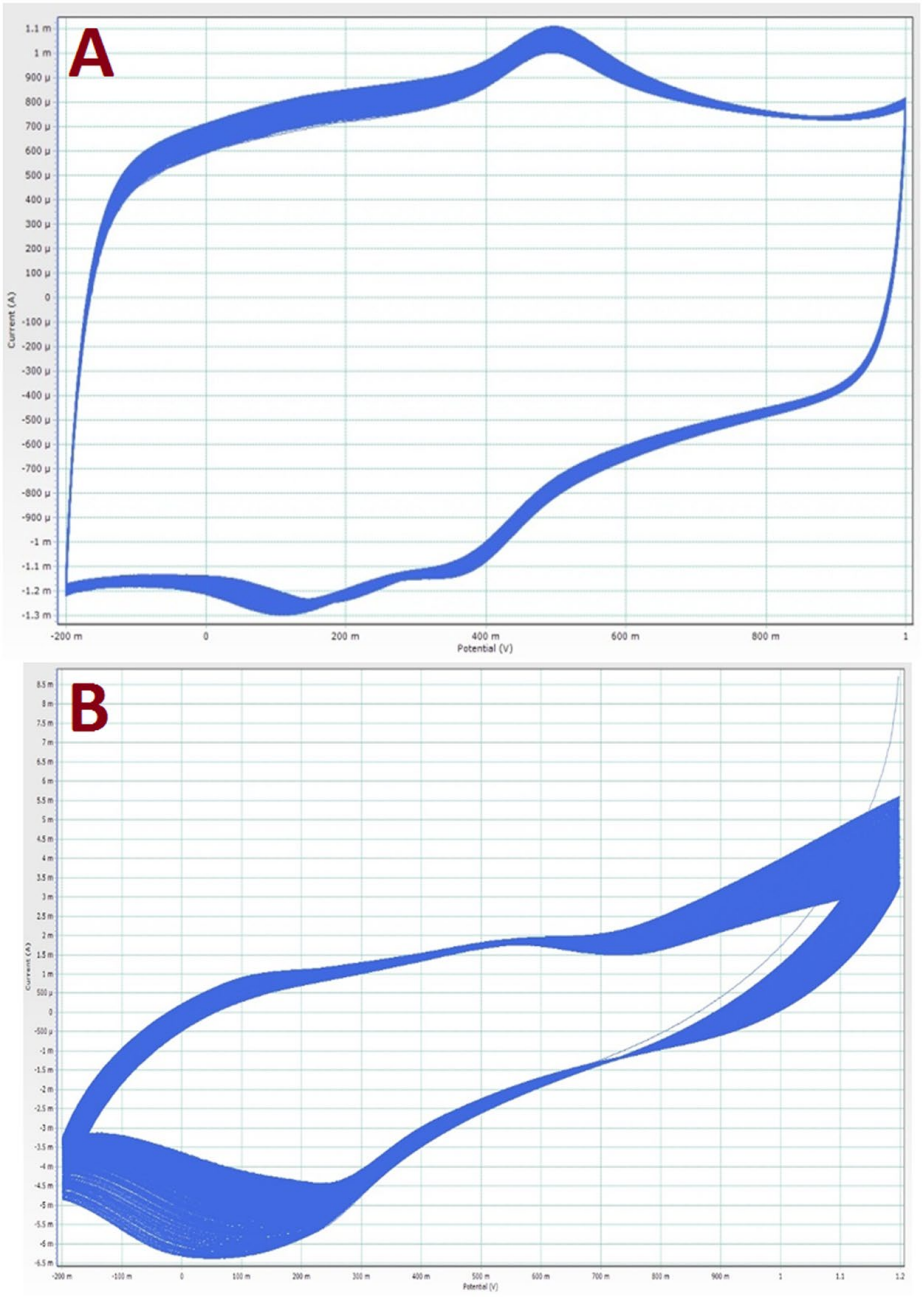
Screen shots for 1000 cycles in 0.5 H_2_SO_4_ for the synthesized FeNi-N-Gr; (**A**) and Pt/C; (**B**).

Stability in the acid medium is the most important advantage of the precious metals and is also a main constraint facing the use of the pristine transition metals. Fast dissolution in acidic solutions is the first concern of transition metals. However, the alloy structure provides novel physicochemical characteristics. For instance, especially at a relatively high nickel content, iron-nickel alloys exhibit high corrosion resistance in acidic media^36,37^. The stability of the introduced and the precious (Pt/C) electrodes was first investigated by cyclic voltammetry analysis for 1,000 successive cycles. Figure 4 displays a comparison between the 4^th^ and 850^th^ cycles of the introduced FeNi-N-Gr (Fig. 4A) and Pt/C (Fig. 4B). Moreover, screen shots for all of the data of the two electrodes can be found in Fig. 5. The obtained results indicate better stability for the introduced electrode compared to Pt/C. The thermodynamic potential of oxygen reduction reaction (1.23 V vs. NHE at S.T.P) is so high that the Pt electrode cannot remain pure. Therefore, platinum undergoes oxidation, which changes the surface properties based on the following reaction:

1 $${\rm{P}}{\rm{t}}{\rm{+}}1{\rm{/}}2{{\rm{O}}}_{2}\to {\rm{P}}{\rm{t}}{\rm{{\rm O}}}\quad \quad \quad \quad {{\rm{{\rm E}}}}^{{\rm{0}}}={\rm{0}}.88{\rm{V}}.$$

**Figure 6:**
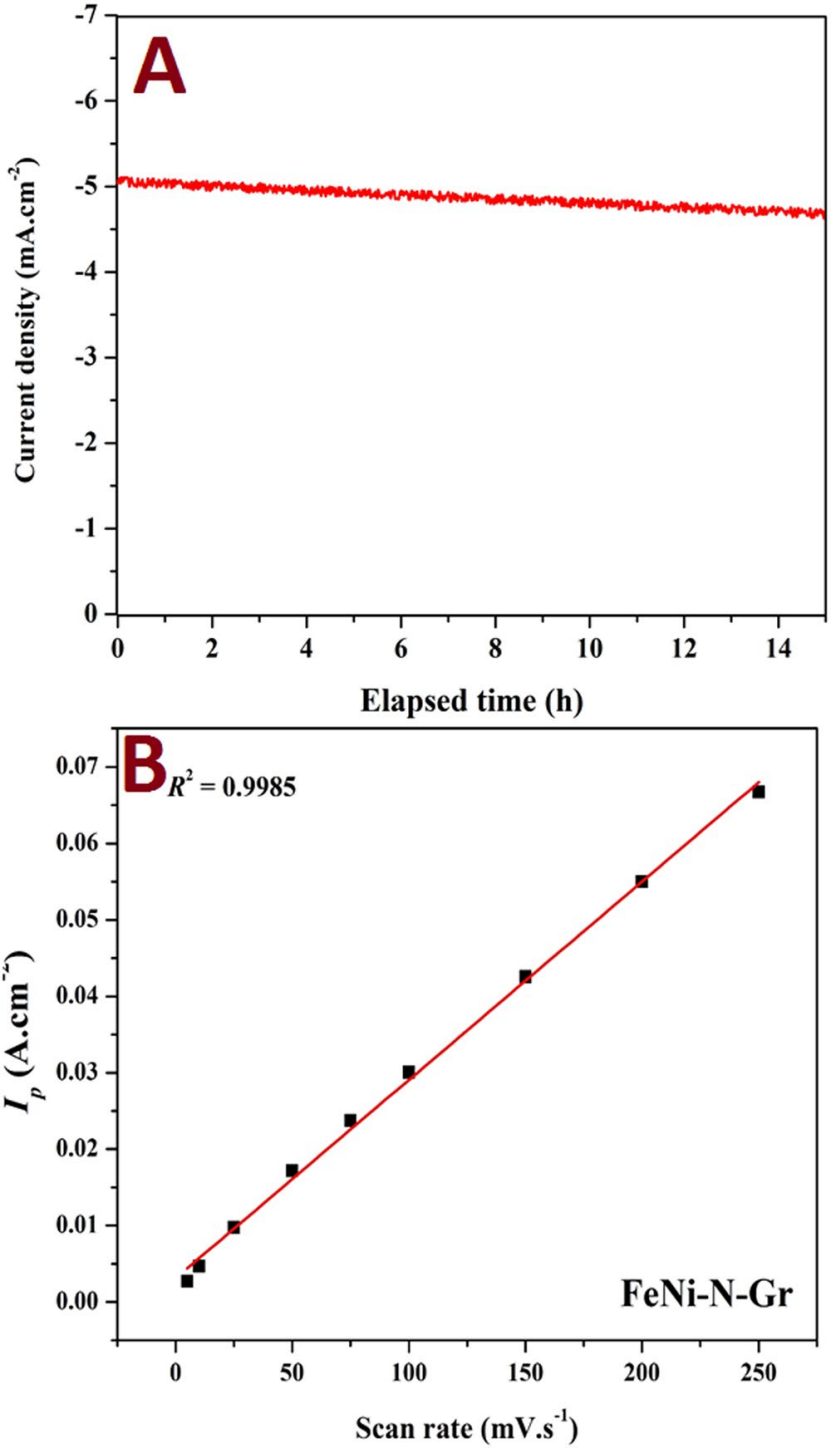
(**A**) Chronoamperometry test for the introduce nanocomposite in 0.5 H_2_SO_4_ at room temperature and (**B**) Peak currents versus scan rate for the introduced FeNi-N-Gr electrode in 0.5 M H_2_SO_4_ at room temperature.

Thus, in the presence of oxygen, the surface of platinum is a mixture of PtO and Pt. Consequently, due to formation of PtO, steady-state open circuit potential (OCP) of 1.23 V is difficult to be obtained. Instead, the steady-state rest potential of the platinum electrode in the oxygen-saturated solutions is around 1.06 V, a mixed value of the thermodynamic potentials of Pt/PtO and O_2_/H_2_O because the two reactions take place simultaneously^38^. Accordingly, with successive cycles, the performance decreases due to formation of PtO. It is noteworthy mentioning that this finding is supported by other reports^39,40^. Alternatively, the introduced catalyst reveals a distinct stability due to the good corrosion resistance and electronic structure of the FeNi alloy. Besides the multiple cyclic voltammetry analysis, chronoamperometery test has been invoked to investigate the stability of the proposed composite; Fig. 6(A). As shown, the results reflect good stability and consequently supports the aforementioned conclusion about the distinct corrosion resistance in the acid media of the FeNi nanoparticles decorating N-doped graphene sheets.

**Figure 7:**
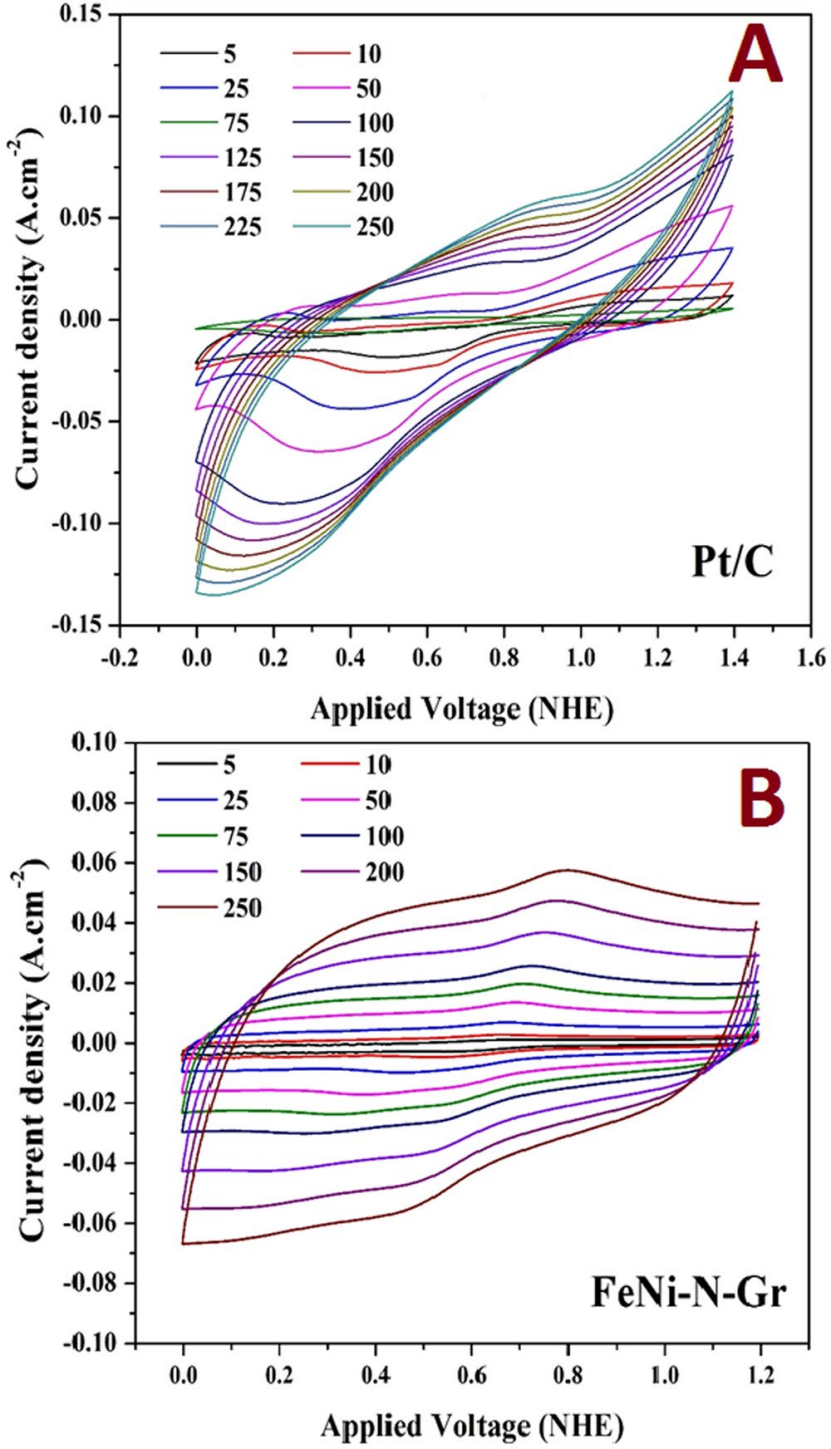
Cyclic voltammetry results for the introduced FeNi-N-Gr and Pt/C electrodes at different scan rates in 0.5 M H_2_SO_4_ at room temperature.

In addition to the current density and onset potential, the number of sharing electrons (*n*) in the ORR is another important factor. Rotating ring-disk electrode (RRDE) analysis is typically invoked to measure the number of the utilized electrons. This technique can give indication about the relative importance of the H_2_O_2_ routes in the overall oxygen reduction reaction process. However, in the literature, for the same material, several *n* values can be found. These different values of the number of transferred electrons can be attributed to the history of the electrocatalytic material which can distinctly affect the ORR rates. For instance, at the ring, in addition to the oxidation of hydrogen peroxides that generates an anodic current, decomposition of H_2_O_2_ without current flow may take place. Moreover, the Pt ring potential can influence the number of electrons, e.g., by varying the relative amount of the formed PtO, and also the synthesis conditions of the investigated material can play a strong role. Therefore, other techniques have been introduced to estimate the number of electrons, such as scanning electrochemical microscopy^41^ and cyclic voltammetry^42^. In the cyclic voltammetry-based procedure, the analysis is performed at different scan rates and the peak currents (*I*_p_) increase linearly with the scan rate that is a typical characteristic of the reaction occurs on the surface of the electrode. The number of the electron can be estimated from the slope according to this equation:

2 $${I}_{p}=\frac{{n}^{2}{F}^{2}}{0.04\,RT}AvS$$

where *n* is the number of the electron transfer, *A* is the electrode active area (0.073 × 10^−4^ m^2^), *v* is the scan rate (mV/s), and *S* is the concentration of the adsorbed oxygen on the electrode surface (here, the maximum value was used as the oxygen solubility in the utilized solution; 3.1 × 10^−4^ M at 20 °C). From the *I*_*p*_ vs. *v* linear relation slope, the number of electrons *n* can be calculated. The voltammograms for the FeNi-N-Gr and Pt/C electrodes are introduced in the Fig. 7. As shown in Fig. 7(B), the linear regression model reveals good fitting (*R*^2^ = 0.998) for the FeNi-N-Gr electrode data. Accordingly, the number of electrons was determined to be 3.89 and 3.465 for Pt/C (linear regression is not shown) and the introduced electrode, respectively.

**Figure 8:**
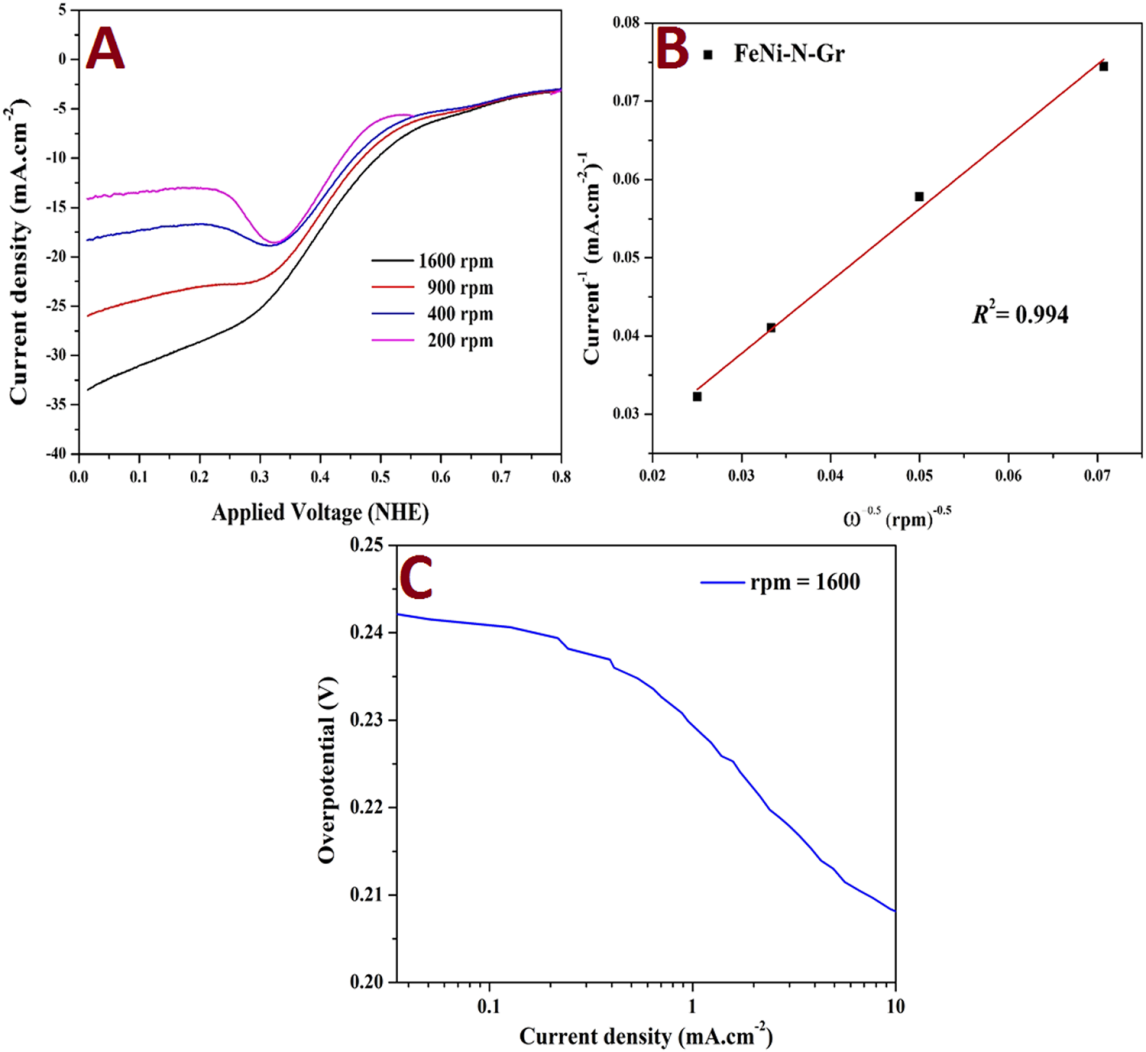
Polarization curve for the oxygen reduction reaction over the introduced FeNi-N-Gr in oxygen-saturated 0.5 H_2_SO_4_ at different rotating rates and sweeping rate 5 mV s^−1^; (**A**) Koutechý-Levich plots for oxygen reduction at 0.1 V (vs. NHE); (**B**) Tafel plot at 1600 rpm (**C**).

For further investigation for the kinetics of the ORR over the introduced catalyst, the polarization curves were carried using rotating disk electrode (RDE); Fig. 8A. As shown, the electrocatalytic current enhances along with the increment of the rotation rate. The increase in the current density can be attributed to an improved catalyst turnover rate for the ORR on the surface of the introduced electrode material. The number of involved electrons in the ORR can be calculated by a Koutechý-Levich graph. Figure 8B shows the obtained Koutechý-Levich plot for the oxygen reduction reaction at a potential of 0.1 V (vs. NHE) on the rotating disk electrode coated with the introduced FeNi-N-Gr in O_2_-saturated 0.5 mol sulfuric acid aqueous solution. Koutechý-Levich equation can be invoked to calculate the number of the involved electros based on this equation:

3 $$\frac{1}{{I}_{lim}}=\,\frac{1}{{I}_{k}}+\frac{1}{B{\omega }^{0.5}}$$

where *I*_lim_ is the experimentally observed limiting current at the selected potential, *I*_k_ is the kinetic current, ω is the rotation rate in rad s^−1^, and *B* can be estimated from this equation:

4 $$B=0.62nFA{C}_{{O}_{2}}{({D}_{{O}_{2}})}^{2/3}{v}^{-1/6}$$

where *n* represents the number of electros, *F* Faraday constant (96486.4 Coulombs), ${C}_{{O}_{2}}$ is the molar concentration of the oxygen (3.1 × 10^−4^ M) at 20 °C, ${D}_{{O}_{2}}$ is the diffusion coefficient of oxygen in water at 25 °C (2.1 × 10^−9^ m^2^.s^−1^), υ is the kinematic viscosity of the solution at 25 °C (1 × 10^−6^ m^2^.s^−1^). As can be distinguished from the value of the linear regression parameter (*R*^2^ = 0.994), the experimental results strongly validate the model, moreover the high linearity obtained suggests first-order of the reaction kinetics toward the concentration of dissolved oxygen^43^. The estimated number of electrons from eq. (4) was 3.57 which supports good activity for the introduced modified graphene electrode and consequently confirm the result obtained from the cyclic voltammetry methodology (eq. 2). Steady-state Tafel plot is the most widely used technique in studying the kinetics of the multistep electrochemical reactions. Tafel expression neglects the mass transport limitations and assumes that the reaction is under kinetic control. Figure 8C shows the Tafel plot for the proposed composite at 1600 rpm. The estimated Tafel slope was 29 mV/decade.

## Conclusion

In summary, graphene sheets were decorated with FeNi nanoparticles by hydrothermal treatment of graphene oxide in the presence of nickel acetate and iron acetate. Moreover, the presence of urea in the reaction medium followed by calcination in an argon atmosphere leads to the incorporation of nitrogen atoms in the graphene skeleton. The produced FeNi-decorated and N-doped graphene can be exploited as a stable and effective electrocatalyst for the oxygen reduction reaction process in acid medium. The high performance was attributed to the good affinity of the metallic nanoparticles and nitrogen atoms to oxygen molecules and hydrogen ions, respectively. Moreover, the good stability in acidic media can be assigned to the alloy structure of the metallic nanoparticles.

## Experimental

### Materials

In the introduced decorated graphene, the used precursors for the metal nanoparticles were iron (II) acetate (FeAc, 99% assay, Sigma Aldrich), and nickel acetate tetrahydrate (NiAc, 99.0% assay, Sigma Aldrich). The reduced graphene oxide was prepared by chemical route using graphite powder (particle size <20 μm), hydrazine monohydrate, hydrogen peroxide, and H_2_SO_4_ (assay 95–97%); these chemicals were purchased from Sigma–Aldrich. All the used chemicals were utilized without further modification. DI water was used as a solvent.

### Procedure

The graphene was prepared chemically from reduction of exfoliated graphene oxide (GO). Typically, GO was synthesized from natural graphite powder by a modified Hummer’s method^26,44^. Briefly: treated twice by 5% HCl five grams of graphite was placed in ice bath concentrated H_2_SO_4_ (130 mL). Later on, 15 g of KMnO_4_ was added gradually to the mixture under zero Celsius temperature condition with stirring for 2 h. Then, distilled water was added to the mixture which results in increasing the temperature to 98 °C. After that the mixture was cold to room temperature, and H_2_O_2_ (50 mL, 30 wt. %) was added, the mixture was kept under stirring for 24 h. Later on, the synthesized GO was separated by filtration under vacuum and washed by10% aqueous HCl several time and then dried at 50 °C. NiFe alloy nanoparticle-decorated and N-doped graphene was synthesized from mixing 0.5 mM iron (II) acetate and 0.5 mM nickel (II) acetate tetrahydrate aqueous solutions with 250 mg of urea (as a source of nitrogen) and stirring for 2 h, followed by ultrasonication for 30 min. As maximizing the nitrogen content in the final product was an important target during the synthesis process, using of urea during preparation of the FeNi-decorated graphene was done (in the reflux step) to incorporate nitrogen atoms within the graphene cycles which effectively increases the nitrogen content during the thermal treatment with melamine^45^. It is worth mentioning that, based on our studies and others, acetate salt was chosen due to the complete reduction during calcination at relatively high temperature under inert atmosphere to produce the corresponding metals rather than the expected metal oxides^46,47^. In another beaker, two hundred mg of the prepared graphene oxide were treated in a microwave oven for two minutes at around 600 W to achieve the thermal exfoliation. The two solutions were mixed and the obtained slurry was then refluxed for twelve hours at 150 °C. The produced slurry was filtered, and the solid material was dried for one day at 80 °C under vacuum. The dried powder was ground with twice as much melamine and calcined under argon atmosphere at 1 atm for four hours at 750 °C. High calcination temperature was chosen to ensure complete reduction of graphene oxide to graphene and avoid formation of metal oxides^46^. Both urea and melamine were used as nitrogen precursors to enhance the nitrogen content in the final product.

### Characterization

Information about the phase and crystallinity was obtained by using Rigaku X-ray diffractometer (XRD, Rigaku, Japan) with Cu Kα (λ = 1.5406 Å) radiation over Bragg angle ranging from 10 to 80°. Normal and high resolution images were obtained with transmission electron microscope (TEM, JEOL JEM-2010, Japan) operated at 200 kV equipped with EDX analysis. The electrochemical measurements were performed on a VersaSTAT 4 (USA) electrochemical analyzer and a conventional three-electrode electrochemical cell. A Pt wire and an Ag/AgCl electrode were used as the auxiliary and reference electrodes, respectively. All potentials were quoted regarding to the Ag/AgCl electrode. Glassy carbon electrode was used as working electrode. Preparation of the working electrode was carried out by mixing 2 mg of the functional material, 20 µL Nafion solution (5 wt%) and 400 µL isopropanol. The slurry was sonicated for 30 min at room temperature. 15 µL from the prepared slurry was poured on the active area (0.073 cm^2^) of the glassy carbon electrode which was then subjected to drying process at 80 °C for 20 min. Cyclic voltammetry measurements were carried out in 0.5 M H_2_SO_4_ solution and the sweep potential range was adjusted from −0.2 to 1.0 V [vs. Ag/AgCl].
